# Case of a Woman Bitten by a Viper

**Published:** 1804-06-01

**Authors:** John Moodie


Medical and Physical Journal.

VOL. XI.] JUNE 1, 1804. [NO. LXIV.

*Printed for R. PHILLIPS, by W. Thorne, Red Lion Court, Fleet Street, London.*

### CASE OF A WOMAN BITTEN BY A VIPER,

### BY JOHN MOODIE, M.D.

CATHARINE BISHOP, aged about 70 years, living at Kelston, four miles and a half from Bath; in the month of April, 1801, whilst gathering violets on a sunny bank, was twice bitten on the back of her hand by a viper. The first time, not having observed the serpent, and thinking she had only been pricked by a brier, having sucked the blood from the wound, she went on picking violets as before. The viper immediately after sprang at her, and pierced the large vein between the middle fingers of the right hand with its poisonous fangs, secured its hold for some time, and it was with difficulty she shook it off, and killed it on the spot with her foot.

The pain was at first extremely acute, and in less than half an hour this person was seized with faintness, sickness at stomach, and vomiting. In this situation she was found by some neighbours, who carried her home, and gave what assistance they could. Towards the evening the wound became livid, the arm was much enlarged, and the skin had a yellowish hue. The infection soon after reached her throat, deglutition was greatly impeded, and she was very restless during the night.

The viper, which had been carried home, was broiled, and the part bitten anointed with its fat, but without giving the smallest relief to the patient. It would, indeed, be a desirable circumstance, if the same animal that produced the poison, should also afford an antidote to destroy its effects. Unfortunately, however, we have not hitherto had any clear proof of the truth of this popular opinion, upon which we can with certainty place our reliance.

The symptoms becoming more alarming, attended with inflammation and fever, the woman was visited about the close of the evening by a neighbouring apothecary, who thought thought that little could be done, he therefore only ordered some olive oil to be given internally, and applied to the wound, with fomentations to the arm, and the body to be kept open; but still the patient received no benefit.

On the morning of the second day, the swelling encompassed her whole breast, and even extended to the bottom of the true ribs, respiration was extremely difficult, the pulse low, quick, and interrupted.

The third day, she appeared in great danger, the swelling having extended over the lower part of the abdomen, and her body was considerably increased beyond its natural size; breathing short and laborious, and her speech was inarticulate.

In this situation the apothecary again saw the patient, but recommended nothing farther, as he supposed that no chance of her recovery remained; the miserable sufferer had not slept for forty-eight hours.

I was now consulted by a relation of this woman, whom I informed that there was no doubt of recovery, if she could swallow the medicine intended to be administered to her; and the person having promised to attend punctually to my directions, I ordered two ounces of *aqua kali puri, olim lixivium saponarium*, a tea spoon full to be taken immediately, in about two ounces of water, or any other convenient vehicle, and repeated every three or four hours, till the patient found relief, and afterwards at longer intervals. The *linimentum ammoniæ fortius* was also directed to be applied to the wound, and frequently to the external parts principally affected.

In less than an hour after the lixivium had been administered, the patient appeared much relieved, the swelling gradually decreased, respiration was less obstructed, and she was more composed. A tea spoonfull of the medicine was then repeated every six hours, and the happy result was, that in three or four days from the first taking the lixivium, she was perfectly restored to health, and a week after came to Bath to return thanks for my advice, and the same day returned to Kelston on foot. At that time she had no pain or uneasiness whatever remaining, except a slight degree of hardness round the edges of the wound. On examination of the part bitten, I could discover the marks of four fangs, two on each side; and from the distance they were asunder, I should have supposed it to have been a large viper.

While employed in the medical department with the king's forces in the East Indies, from 1781 to 1788, my professional professional situation with the army gave me frequent opportunities of observing the symptoms peculiar to, as well as the various means at that time recommended by authors, and generally adopted, in order to afford relief to persons unfortunately bitten by venomous snakes.

The medical practitioners of the French, and other European nations, who had settlements in India, were in the habit of giving arsenic, in the form in which that dangerous medicine had been long administered, and too often with fatal effects by the natives of the country. The volatile alkali has been much depended upon by some, as a specific against the poison of the viper. Many years ago it was brought into general practice by the recommendation of M. de Jussieu, and in several instances is known to have been sufficiently efficacious, by stimulating the fibres and preserving that irritability which the effects of the poison tend to destroy.

The surgeon-major to the French forces on the coast of Coromandel informed me, that he had not employed the volatile alkali, or the lunar caustic, in resisting the disorder occasioned by the poison of serpents; but that he had generally ordered from twelve to sixty drops of the *spiritus volatilis succinatus*, long known by the name of *eau de luce*, with considerable success internally, and applied to the wounds of persons bitten. That during the campaign of 1782, he had twelve patients attacked with the cramp, all of whom he cured by the *spiritus salis ammoniaci cum calce*, or caustic volatile alkali. When the disorder fell under his care, he gave ten or twelve drops of this medicine in half a glass of water; soon after which a perspiration broke out; this he kept up by a decoction of cinnamon or sage, of which he ordered the patient to drink plentifully, keeping him to a strict regimen. The next day the patient was generally easy, and frequently cured; if the first dose did not operate, he gave a second or third, in the same manner.

Spasmodic diseases endemial to hot countries, are not only prevalent at particular seasons, but are the most fatal disorders to which Europeans are subject after their arrival in India; and exceed all of the kind ever known in cold climates, in the suddenness of their attack, extreme violence, the painful symptoms which they produce, their duration, and finally, if not speedily relieved by medical assistance, by their fatal termination.

In the cure of this complaint from the authority above mentioned, I was induced to make trial of the volatile alkali, alkali, and generally with success, but given in much larger doses than those recommended by the French surgeon. I have, however, always found the most beneficial effects from the warm bath, a liberal use of opium in a liquid form, joined with a strong and active cordial, in small quantities, and frequently repeated.

The volatile alkali, acting as a powerful stimulus, is often prescribed in diseases where the pulse is languid, and the patient in danger of sinking; it is also of great utility in promoting the secretions, especially by the skin. And it seems principally owing to this, and their stimulating quality, that those salts have acquired the reputation of being specific remedies against the bad effects of the bites of serpents, and other noxious animals.

In what manner the poison of the serpent occasions such dreadful symptoms, and sudden death upon animal bodies, is a circumstance which remains to this hour involved in obscurity and doubt. It is, however, an important fact, and known to ancient writers, (though contrary to the authority of Dr. Mead) "that the venom of serpents produced its effects in consequence of a wound, and thro' the medium of the blood."

It has also been a general opinion, that the bite of the viper, and other noxious serpents, will sometimes occasion jaundice. This effect, says Dr. Mead,* is owing to spasms obstructing the biliary ducts, in the same manner as colic pains produce this disorder. But, it may be doubted, whether colic pains ever produce jaundice of themselves, without the assistance of *calculi,* or other exciting causes obstructing the biliary passages. Dr. Simson† observes, from several experiments, that jaundice in this case arises from the operation of the poison acting immediately on the blood, and producing such a change in it, without causing any obstruction in the ducts, or any regurgitation of bile from the liver.

Fernelius‡ appears to have had the same notion of it, when, speaking of *Viperæ demorsum aut epotum venenum,* as causes which bring on jaundice, says, *Quorum vi sanguis totus pristinam amittet puritatem, et in citrinum ac biliosum humorem corrumpitur, qui omnia pervadens cutem inficit atque labefactat.*

The

* Treatise on Poisons.

† See his Essay on the Jaundice, Med. Essays and Obs. vol. 1, edit. 4.

‡ Patholog. lib. vi. cap. 8.

The Abbé Fontana* has clearly demonstrated, from a variety of experiments on different animals, that the venom of the viper is perfectly innocent when applied to the nerves only; that it produces in them no sensible change, and that they are incapable of conveying the poison to the animal. On the other hand, he has shewn, that it acts immediately on the blood, and that, through the medium of the circulation, it destroys the irritability of the muscular fibres, and thereby occasions death.

Upon this view of the subject, it would not indeed seem difficult to comprehend in what manner the peculiar quality of the venom acts, so as to cause sudden death. It has been confidently reported by Fontana, and other naturalists that no instance of the bite of a viper proving fatal in Europe, can be ascertained; a happy circumstance were this true. If in Europe, at certain seasons, the poison of the viper should happen to be immediately thrown into a large vein running on the surface of the body, and the animal be of a size, so as to introduce a sufficient quantity of venom, (as occurred in the case of the woman now described) it will be rapidly carried to the vital parts; and even in the human body, may render the use of the most powerful medicines ineffectual.

It is, however, pleasing to reflect, that, of the many serpents supposed to be venomous, few are really so; and even these do not, in every instance, inflict a fatal wound. And though death sometimes happens from the bites of the European viper, as well as the Indian serpents, yet, recovery from the wounds of those supposed to be most poisonous is not uncommon.

The distinction between poisonous and innocuous serpents was long ago described by Dr. Gray, in the Philosophical Transactions for 1789, vol. lxxix; where he says, that he had examined one hundred and fifty-four species of serpents, of which number twenty-six only appeared to be venomous.

The effects produced by the bite of a viper, or other noxious animals, are so alarming and fatal, that we cannot wonder to find they have excited the attention of the most eminent physicians in all ages, to investigate the cause, and find out a powerful remedy for so dreadful an evil. But it would be endless to enumerate the various medicines which from remote antiquity, have been successively imposed

* See Fontana, Traite sur le Venin de la Vipere.

imposed upon the credulity of mankind, as specifics against the poison of serpents.

Arsenic has been long used by the natives of India, as a principal ingredient in the celebrated Tanjore pill; the composition of which is, white arsenic, pepper, quicksilver, the roots of velli-navi, (a poisonous vegetable peculiar to the Malabar coast) the roots of the neri-visham, and the kernels of nervalam, both drastic purgatives. The quicksilver being rubbed down in a mortar, with the juice of wild cotton, till the globules entirely disappear, and the other ingredients carefully powdered, the whole is beaten up into a mass for pills with the above juice.

I cannot help looking upon arsenic as a dangerous remedy, even when administered with every caution possible; and although it may be well adapted to counteract the effects of the poison, yet, as it often produces dangerous symptoms, and if used with freedom might occasion death, I was therefore deterred from ordering it.

Although many years have elapsed since Fontana published his experiments, respecting the internal use of the *lunar caustic,* now called *argentum nitratum;* yet, notwithstanding the advantage said to have arisen from this preparation of silver in the *nitric acid,* it is surprising that it has not hitherto excited more general attention. The *argentum nitratum* may be taken two or three times in the day, in the quantity of half a grain dissolved in two ounces of distilled or rain water, (otherwise the caustic will be in part decomposed, which will be evident by the white cloud forming in the solution); its use may be continued for several days with the greatest safety. The effects it produces, are generally heat in the stomach and breast, and after a time, tenderness in the gums, with a disposition to bleed, but without that swelling and pain attending the use of the oxyds of mercury.

Fontana mixed the poison with the lunar caustic, which he applied to a wound, and found that the venom was rendered perfectly innocent, while the corroding power of the caustic was diminished. He wounded a variety of animals with poisonous teeth, scarified the wounds, and washed them with a solution of the same caustic in water; by which means the lives of the greater number of animals were saved, though he knew they were such as were most easily to be killed by the poison, and the death of others was retarded. He in like manner tried a weak solution of the same medicine internally with great success; hence he congratulates himself in seeing his labours at length length rewarded by the discovery of a true specific remedy for the bite of the serpent.

From the above account, we should have imagined the lunar caustic to be capable of curing every case of this kind, in which it was administered; but I am sorry to say, that after repeated trials made in several cases, I did not find it deserved the praises bestowed upon it by Fontana; and many practitioners have assured me, that they have been greatly disappointed in their expectation of its powers. And there are instances of *rabies canina,* terminating fatally after the use of the lunar caustic, and salivation by mercury. Although the utility of the several methods have not yet been fully established, there is reason to hope, from the spirit of investigation which now prevails, that some solid advantage will at length be derived for the benefit of the public.

When persons are unfortunately bitten by animals whose venom is highly deleterious, it is the duty of the practitioner to stop the progress of the disorder as speedily as possible; and this can only be done by a medicine, the operation of which is quick and effective. In various cases which came under my own direction, five of whom were bitten by the *cobra de capello,* the *cerastes,* and other serpents known to be highly poisonous, four recovered by the use of the lixivium saponarium.

When called to the patient immediately after the accident, my first attention was to apply a ligature* between the part bitten and the heart, to prevent the poison from diffusing itself over the body by means of the returning venous blood. With respect to medicines, I gave the preference to the lixivium saponarium, or the caustic volatile alkali, either of which, if timely administered, has been found sufficient to obviate the dangerous tendency of the poison. Of the above medicines, I ordered sixty or seventy drops, to an adult, in about two ounces of water, which was repeated every three or four hours, till the patient was relieved.†

Whatever may be the nature of the poison of the serpent,

* Though the use of the ligature has been considered by Fontana of no utility; yet, in compliance with custom, if this practice can give the patient a chance of recovery, it ought not to be neglected.

† An experienced practitioner will observe that mercury, the nitric acid bath, and many of the most powerful articles of the Materia Medica, are still left untried; but whatever measures are used under such circumstances, they must be prompt and efficacious.

pent, or of the *modus operandi medicaminum,* we know that the two fixed alkaline salts unite readily with acids, and change them into a mild neutral. Hence, if by accident or otherwise, any of the strong mineral acids should fall on any part of the human body and begin to corrode and give pain, the immediate application of the *aqua kali,* or a solution of any of these alkaline salts, will destroy the causticity of the mineral acids and prevent them from doing farther mischief. Or if any person should accidentally swallow any of the mineral acids, or hydrargyrus muriatus, or any other corroding salt, which an alkali will decompose, a speedy exhibition of a solution of the alkaline salts, in proper doses, afford the most likely means of relief and of preventing fatal effects.

